# A robust hybrid data driven approach to model biochar yield in terms of biomass pyrolysis

**DOI:** 10.1186/s40643-026-01058-9

**Published:** 2026-04-29

**Authors:** Kusum Yadav, Lulwah M. Alkwai, Shahad Almansour, Mehrdad Mottaghi

**Affiliations:** 1 College of Computer Science and Engineering, University of Ha′il, Ha′il, Kingdom of Saudi Arabia; 2 Applied College, University of Ha′il, Ha′il, Kingdom of Saudi Arabia; 3 Faculty of Chemistry, Kabul University, Kabul, Afghanistan

**Keywords:** Biochar yield, Data analysis, Optimization, Decision tree, Biomass pyrolysis

## Abstract

Biochar yield prediction plays a critical role in optimizing pyrolysis processes and advancing sustainable biomass utilization. This study introduces a hybrid machine learning framework that integrates Decision Tree models with four optimization strategies including Tabu Search, Ant Colony Optimization, Evolutionary Strategies, and Batch Bayesian Optimization (DT-BBO). A curated dataset of 211 samples was preprocessed using Leverage outlier detection and normalization to ensure model robustness. Among all tested models, the DT-BBO approach achieved the highest accuracy, with an R^2^ of 0.98, an MSE of 1.9, and an AARE% of 2.2%, outperforming the other optimization techniques. SHAP analysis revealed that pyrolysis temperature, residence time, and ash content were the most influential parameters governing biochar yield. Comparative benchmarking against previously published models confirmed the superior predictive capability and stability of the proposed framework. The results demonstrate that the DT-BBO model offers a scalable, interpretable, and high-performing solution for biochar yield prediction, contributing to methodological innovation and sustainable biomass valorization.

**Graphical abstract:**

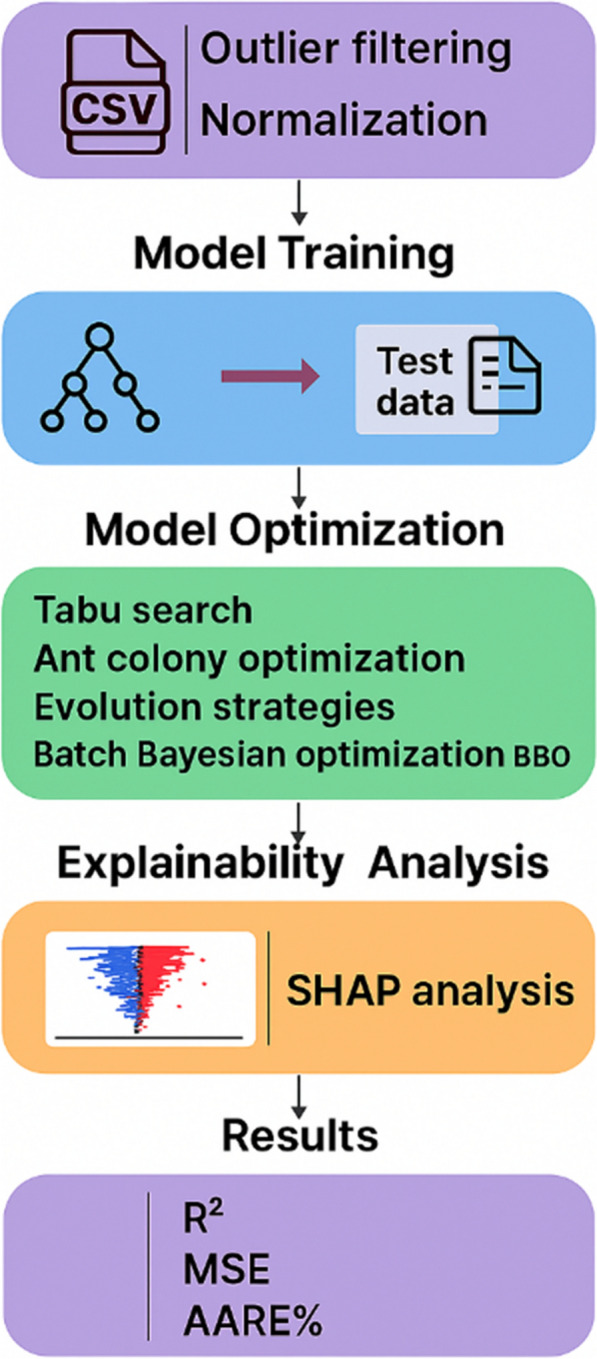

## Introduction

Biomass is one of the world’s most abundant renewable resources, generating about 105 billion metric tons of carbon annually from both terrestrial and marine ecosystems. In contrast, heavy global dependence on finite fossil fuels has led to major environmental issues, including greenhouse gas emissions and global warming (Chun et al. 2021; da Silva Medeiros et al. 2025; Hu et al. 2026; Ibitoye et al. 2024; Zhu et al. 2025). As a result, renewable energy sources have become increasingly important, with biomass standing out for its sustainability, accessibility, and lower net carbon emissions. Within this context, biochar has gained renewed attention due to its diverse applications in agriculture, energy production, and waste management (Ge et al. 2023; Guo et al. 2025; Ibitoye et al. 2024; Puri et al. 2024; Rahic et al. 2026).

The efficiency and characteristics of biochar production are governed by two main factors: the operational parameters of the pyrolysis process and the type of biomass feedstock (Birhanu et al. 2025; Manyà et al. 2018; Wallace et al. 2019; Zhou et al. 2024). Operational factors, including peak temperature, residence time, heating rate, and pressure, are particularly influential in determining biochar yield and quality (Park and Jang 2012). In contrast, higher temperatures tend to increase the release of volatile matter, which reduces the biochar yield but enhances its aromaticity and stability (Kang et al. 2025; Li et al. 2025; Paudel et al. 2025). Similarly, residence time directly affects carbonization efficiency. Longer residence times promote a more complete thermal breakdown of biomass, which increases the proportion of fixed carbon in the resulting biochar (Herath and Janaranjana 2025; Xiao et al. 2024; Xue et al. 2025). Other variables, such as pressure and the pyrolysis atmosphere, also influence biomass conversion. While inert gases like nitrogen are traditionally used to prevent oxidation, recent research indicates that substituting these with CO_2_ from residual flue gases can improve cost efficiency without significantly compromising biochar properties related to carbon sequestration (Franzon 2025; He et al. 2025; Li et al. 2025; Wang et al. 2024).

Recent studies have demonstrated the growing role of ML in calculating biochar yield and biomass properties, with particular emphasis on explainable approaches such as SHAP analysis (Abdelfattah et al. 2025a, b; Birhanu et al. 2025; Hou et al. 2025; Le et al. 2023). These works highlight the importance of integrating interpretability into predictive models, ensuring that statistical accuracy is complemented by mechanistic understanding of pyrolysis processes (Nguyen et al. 2024). Incorporating such findings situates the present study within the broader research landscape, underscoring both the methodological innovation and practical relevance of the proposed DT-BBO framework (Nguyen et al. 2024a, b).

A critical gap in the literature is the absence of studies that jointly examine multiple metaheuristic and probabilistic optimization algorithms applied to a single predictive model, while simultaneously providing interpretable insights into parameter influence. This gap limits both methodological advancement and practical applicability, underscoring the need for a comprehensive, optimization-driven, and interpretable framework for biochar yield prediction.

This study demonstrates the value of machine learning for optimizing biochar production, a material with important environmental and agricultural benefits. The workflow begins with assembling a high-quality dataset from published biomass-pyrolysis studies, covering 14 chemical, physical, and operational parameters. After preprocessing the data using Leverage outlier detection and normalization, several machine-learning models are trained and evaluated using hyperparameter tuning and fivefold cross-validation. Model accuracy is assessed through R^2^, MSE, and AARE%, and SHAP analysis is applied to interpret feature importance, revealing how variables such as ash content, pyrolysis temperature, and residence time influence biochar yield.

**Fig. 1:**
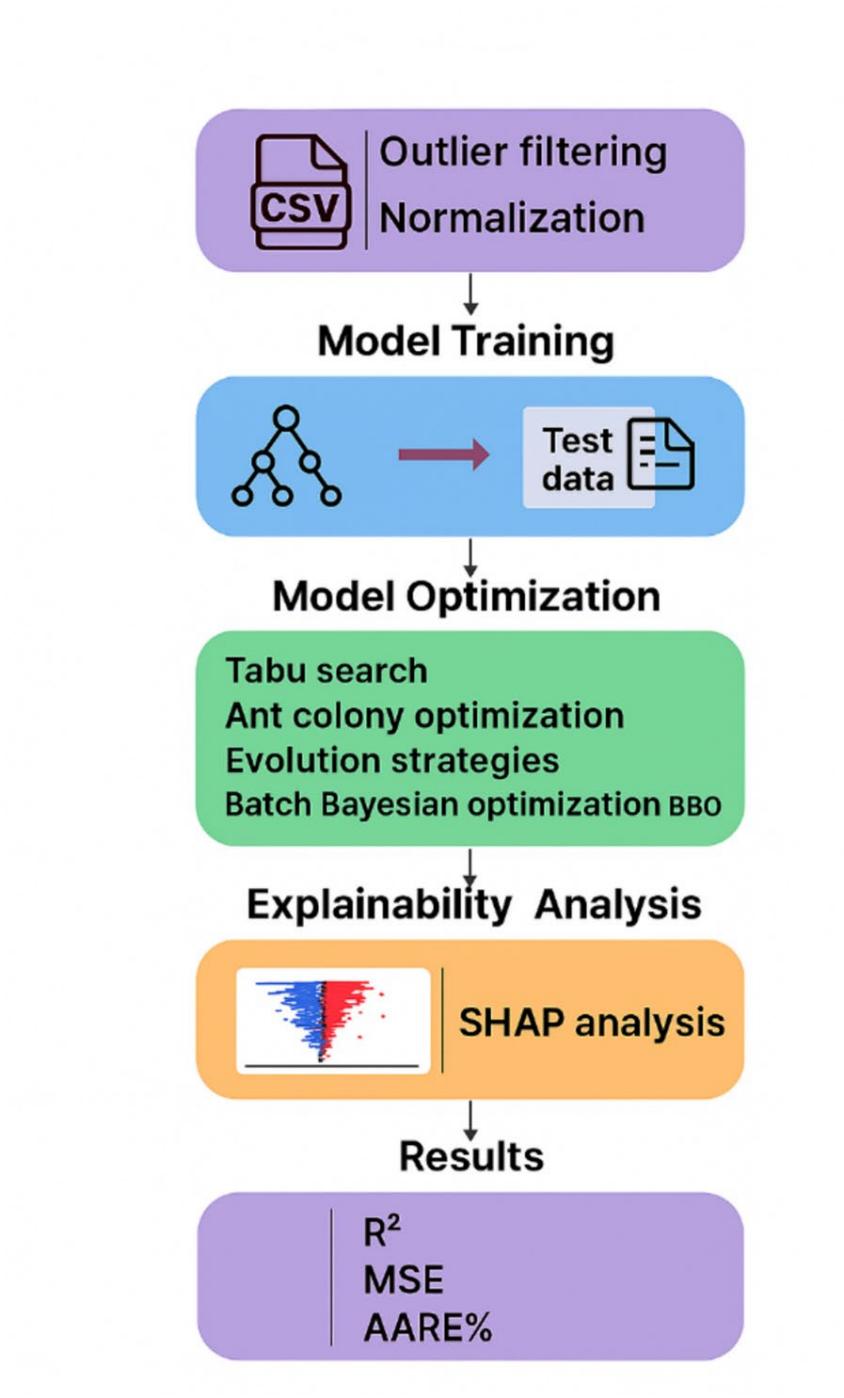
Schematic representation of the study workflow

To enhance predictive performance, the study integrates Decision Tree models with four distinct optimization strategies, Tabu Search, Ant Colony Optimization, Evolutionary Strategies, and Batch Bayesian Optimization. This comparative design captures the strengths of different search paradigms and identifies DT-BBO as the most effective approach. By combining rigorous preprocessing, multiple optimization algorithms, and SHAP-based interpretability, the research offers a unified and transparent framework that advances beyond previous machine-learning studies. The result is a scalable, accurate, and interpretable model that strengthens both methodological innovation and practical understanding of biochar formation. The overall workflow is given in Fig. 1.

## Machine learning methods background

### Decision tree

Decision tree algorithms build a hierarchical, tree-like model by repeatedly splitting a dataset into smaller and smaller segments. This process starts with the entire dataset at the root node and works its way down. At each node, the algorithm evaluates different features to find the one that best divides the data. The goal is to create "pure" child nodes, where most of the data points belong to the same category. This selection of the best feature to split on is guided by specific metrics, such as Gini impurity, information gain, or variance reduction, which quantify how effective a particular split will be (Zhuang, Liu, Singh, Shang, & Gao, 2024).

The splitting process doesn't continue indefinitely; it stops when a pre-defined stopping condition is met. These conditions are crucial for preventing the tree from becoming too complex and fitting the training data too closely, a problem known as overfitting. For instance, the algorithm might stop when it reaches a set maximum depth or when all of the samples in a particular node belong to the same class, making it a "pure" leaf node.

For classification tasks, the algorithm uses specific metrics to assess the quality of a split. Entropy is a key measure that quantifies the impurity or randomness of the data within a node. The formula for Entropy is (Sepúlveda-Fontaine and Amigó 2024):

1 $$ Entropy = - \sum\limits_{i = 1}^{c} {p_{i} \log_{2} \left( {p_{i} } \right)} $$

Here *p*_*i*_ is the proportion of samples belonging to class i, and c is the total number of classes. A node with a mix of classes has high entropy, while a pure node has an entropy of zero.

This concept of Entropy leads to another critical metric: Information Gain (IG). Information Gain measures the reduction in entropy after a dataset is split on a feature. The algorithm calculates the IG for each potential split and chooses the feature that provides the highest gain. The formula for Information Gain is (Kumar et al. 2025):

2 $$ IG = Enthropy\left( {parent} \right) - \sum\limits_{k - 1}^{m} {\frac{{n_{k} }}{n} \cdot Entropy\left( {child_{k} } \right)} $$

In this formula, *n*_*k*_ is the samples number in the k^th^ node, n is the total samples, and m is the splits.

While decision trees are powerful, they are prone to a major drawback: overfitting. If a tree is allowed to grow too deep, it can memorize the training data's noise rather than learning its underlying patterns. To counter this, techniques like pruning (removing branches from the tree) and setting constraints are used. Decision trees focus on reducing the variance of the output values in the leaf nodes, typically by minimizing the Mean Squared Error (MSE).

### Evolutionary strategies (ES) optimization

These techniques are a class of optimization algorithms that draw inspiration from natural evolution, specifically designed for continuous optimization issues. The core principle contains the iterative improvement of a population of candidate solutions across sequential generations (Peng et al. 2024).

The evolutionary cycle commences with the initialization of a population, where each individual is represented by a solution vector and a corresponding set of strategy parameters that govern the mutation strength. Subsequently, in each generation, a new set of individuals, known as offspring, are generated. This is primarily achieved through mutation, a process that introduces random alterations to both the solution vectors and their strategy parameters. Additionally, recombination may be employed to merge genetic information from several parent individuals to generate new offspring.

Following the creation of offspring, a selection phase is conducted to determine the individuals that will constitute the parent population for the next generation. Two principal selection schemes are commonly utilized: the (μ + λ)-ES strategy, which selects the best μ individuals from the combined pool of μ parents and λ offspring; and the (μ,λ)-ES strategy, which selects the best μ individuals exclusively from the λ offspring. This selective pressure ensures the progressive improvement of the population over sequential generations.

A hallmark of Evolution Strategies is their self-adaptation mechanism, wherein the strategy parameters evolve alongside the solution vectors. This dynamic adaptation enables the algorithm to autonomously adjust its search behavior, striking a balance between the global exploration of the search space and the local exploitation of promising regions. Advanced variants, such as Covariance Matrix Adaptation Evolution Strategy (CMA-ES), further optimize performance by learning the covariance structure of the population's search distribution. The iterative process of initialization, mutation, optional recombination, and selection is repeated until a predefined termination criterion is reached, at which point the best solution identified throughout the procedure is returned as the ending result (Dziwiński & Bartczuk).

### Batch bayesian optimization (BBO)

This approach is an advanced method that improves traditional Bayesian optimization by accelerating the search for the optimal solution. It achieves this by evaluating multiple candidate points at the same time, instead of one by one. It is particularly effective in environments with parallel calculation resources, like high-performance clusters, where it can significantly reduce the total time needed to find a solution. The core advantage of BBO is its ability to speed up the optimization process without sacrificing the careful balance between exploring new areas and exploiting promising ones, a hallmark of Bayesian optimization (Azimi et al. 2012).

Unlike sequential methods, which pick the single best candidate at each step, BBO selects a group of points together. This requires a specialized acquisition function that can account for the addictions and possible overlay of information. Simply selecting the best individual points might lead to redundant evaluations, as the information gained from one point could make another in the same batch less valuable. The acquisition function is designed to prevent this by considering the collective benefit of the entire group (González et al.).

One of the most common acquisition functions for BBO is Parallel Expected Improvement (q-EI). This function extends the concept of Expected Improvement to batches of size q. Instead of calculating the potential improvement for just one state, q-EI approximates the communal expected improvement for whole q points. It does this by considering the joint probability spreading of the points, as predicted by the underlying statistical model (usually a Gaussian Process). By maximizing q-EI, the algorithm ensures that the selected batch is chosen to maximize the total expected gain, making the search more efficient (Azimi et al. 2010).

### Tabu search optimization (TSO)

The Tabu Search Optimization (TSO) is a metaheuristic search algorithm designed to find optimal or near-optimal solutions to complex combinatorial optimization problems. It works by intelligently exploring a solution space, moving from one potential solution to another. Unlike greedy algorithms that might get stuck in local optima, TSO employs a memory-based approach to guide its search. The algorithm's core idea is to systematically explore the solution landscape while strategically avoiding revisiting previously explored solutions that are deemed "tabu" for a certain number of iterations. This aversive strategy allows it to escape local optima and continue its search for a better global solution (da Conceicao Cunha and Ribeiro 2004).

The algorithm maintains a tabu list, which is a short-term memory of recently visited solutions or moves. When the algorithm moves from the current solution to a new one, the move itself is added to the tabu list. Any subsequent move that would lead back to a recently visited state is considered "tabu" and is forbidden. The size of this list, known as the tabu tenure, is a critical parameter that determines how long a move remains forbidden. This mechanism prevents the algorithm from getting caught in cycles and encourages it to explore new regions of the solution space (Repalle et al. 2022).

However, the tabu list isn't an absolute constraint. TSO includes a concept called aspiration criteria, which allows the algorithm to override the tabu status of a move. A common aspiration criterion is to permit a tabu move if it leads to a solution that is better than the best solution found so far. This flexibility ensures that the algorithm doesn't miss out on potentially superior solutions, even if the path to them is currently marked as tabu (Abido 2002).

In summary, TSO is a powerful and flexible optimization technique. It systematically searches for a solution by making a series of moves from a current solution to a neighbor. Its key strength lies in the use of a memory structure (the tabu list) and aspiration criteria to intelligently guide the search. This prevents it from getting stuck in suboptimal solutions, allowing it to explore the search space more thoroughly and effectively (Panigrahy et al. 2024).

### Ant colony optimization (ACO)

Ant Colony Optimization (ACO) is a metaheuristic inspired by the foraging behavior of real ants. It is used to find solutions to complex optimization problems, such as finding the shortest path in a graph. The fundamental idea behind ACO is that a group of simple, individual agents (simulated ants) can collectively solve a difficult problem through indirect communication. This communication happens through a chemical substance called pheromone. When an ant finds a good path, it deposits more pheromone, leaving a trail for other ants to follow. Over time, the most efficient paths will accumulate more pheromone, attract more ants and reinforce those paths as the optimal solution (Dorigo and Stützle 2018).

The algorithm begins by placing a group of virtual ants on the nodes of a problem graph. These ants then move from node to node, making choices based on two main factors: the pheromone trail intensity and a heuristic desirability of the next step. The heuristic information is specific to the problem; for example, in the Traveling Salesman Problem, a heuristic might favor moving to closer, unvisited cities. Ants are more likely to choose paths with higher pheromone levels and more desirable heuristic values, creating a positive feedback loop (Dorigo et al. 2007).

As an ant travels, it leaves behind a pheromone trail on the edges it crosses. The amount of pheromone deposited is often related to the quality of the solution found by that ant. For example, an ant that completes a very short tour in the Traveling Salesman Problem would deposit a larger amount of pheromone than an ant that completes a longer tour. This process reinforces the better paths, making them more attractive to subsequent ants (Awadallah et al. 2025).

The pheromone trails are not permanent. Over time, the pheromone on all edges evaporates at a certain rate. This is a crucial aspect of the algorithm, as it prevents the system from prematurely converging on a suboptimal solution. Evaporation helps the algorithm forget bad solutions and allows for the exploration of new areas of the solution space. If a path is not chosen by any ants for a while, its pheromone level will decrease, making it less attractive and freeing up the algorithm to consider other options (Akkaya and Közkurt 2024).

In essence, the Ant Colony Optimization algorithm is a powerful tool for solving problems that can be represented as a search for the best path in a graph. It leverages the collective intelligence of a simulated ant colony. The key components are the use of a pheromone-based positive feedback mechanism to reinforce good solutions, a heuristic component to guide the initial search, and a pheromone evaporation mechanism to avoid stagnation and encourage exploration. This combination allows ACO to effectively explore large search spaces and find high-quality solutions to a wide range of combinatorial optimization problems (Blum 2024).

## Data explanation and assessment

### Data explanation

The data for the generated model was based on a widespread literature review related to biochar production, specifically targeting data from studies on biomass pyrolysis (Chaihad et al. 2021; Gurevich Messina et al. 2017; Hatefirad et al. 2022; Li et al. 2022; Mishra and Mohanty 2022; Ozbay et al. 2019; Park et al. 2020; Shahsavari and Sadrameli 2020; Shen and Chen 2023; Wei et al. 2022; Wei et al. 2020; Wu et al. 2022a, b; Wu et al. 2022a, b; Xue et al. 2022; Yang et al. 2020; Yarbay Şahin and Özbay 2021; Zeng et al. 2021; Zhang et al. 2015; Zheng et al. 2017). All 211 data points used in this study were compiled exclusively from peer-reviewed literature on biomass pyrolysis, ensuring that the dataset reflects experimentally validated results across a wide range of biomass types and operating conditions. No proprietary or in-house experimental data were used. The compiled data originates from a broad range of peer‑reviewed studies conducted across Asia (China, India, Malaysia, South Korea), Europe (Spain, Italy, Poland), North America (United States, Canada), and the Middle East. To ensure the quality and credibility of the data, the selected research articles were sourced exclusively from high-impact, peer-reviewed journals. The resulting dataset consists of 14 independent variables and one dependent variable, with the latter being the target for prediction by a range of machine learning models.

The independent variables were organized into three groups. The first includes chemical and compositional attributes of the biomass, its carbon, hydrogen, nitrogen, oxygen, ash, fixed carbon, and volatile-matter contents, as well as the catalyst’s acid-site concentration (mmol/g). The second group captures physical characteristics, represented by the crystallinity index and BET surface area. The third group consists of operational reaction conditions: catalyst-to-biomass ratio, pyrolysis temperature (°C), and residence time (minutes). The dependent variable is the biochar yield, measured as the weight percentage of solid product generated during pyrolysis. Although pyrolysis also produces syngas and various organic compounds, biochar yield is emphasized because of its established importance in agricultural uses such as soil enhancement and food production.

**Fig. 2:**
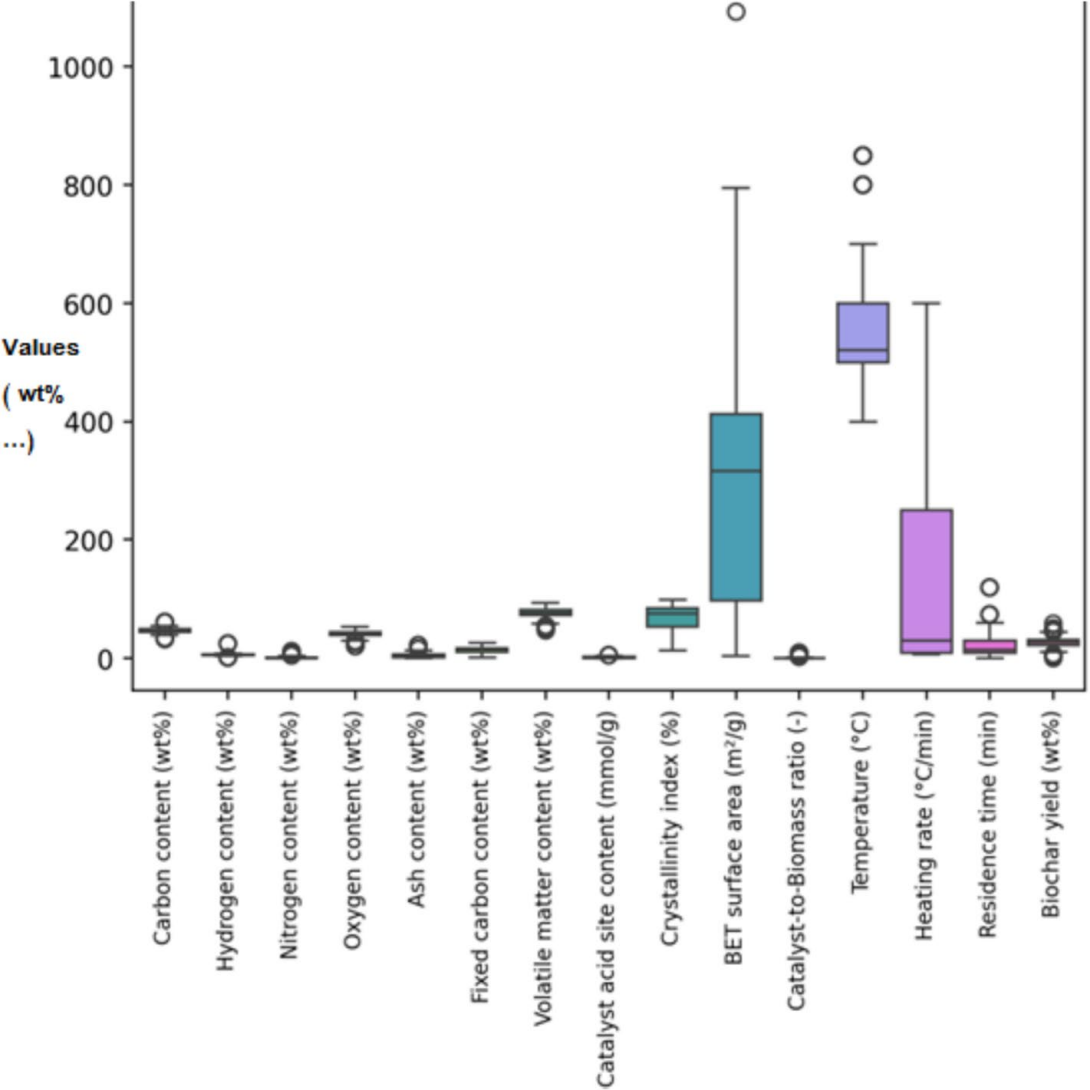
Data points’ box plot analysis

A dataset of 211 data points was employed for this study. The data was partitioned, with 90% designated for training the machine learning models and the remaining 10% reserved for independent testing and validation. The box plot in Fig. 2 visually represents the dataset’s overall distribution.

To enhance the performance and applicability of the predictive models, K-fold cross-validation was employed as a reliable statistical method. This technique involves partitioning the complete dataset into 'K' non-overlapping subsets, or folds. In each of the 'K' iterations, a different fold is held out as the validation set while the remaining K-1 folds are utilized for model training. The final performance of the model is determined by averaging the results across all 'K' validation iterations, which effectively reduces the bias associated with a single, arbitrary train-test split.

**Fig. 3:**
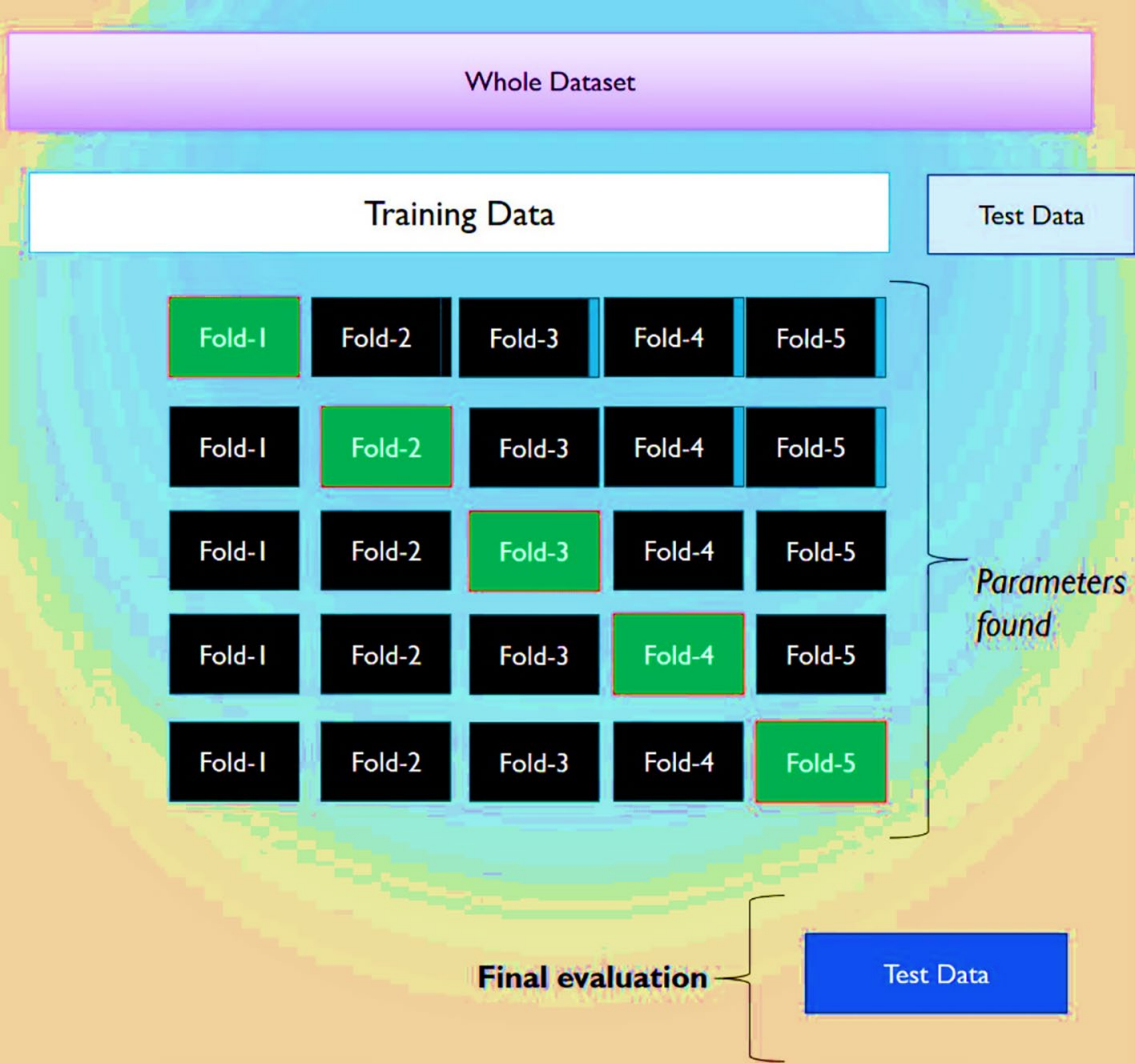
Schematic of the K-Fold Cross-Validation process, detailing the division of the dataset

This approach is particularly valuable for its ability to perform a more accurate valuation of a model's prediction capacity and to detect overfitting. As shown in Fig. 3, the K-fold cross-validation procedure ensures a thorough evaluation of the model's prediction capability in diverse data. For this research, a fivefold cross-validation procedure was implemented for all algorithms during the train step, thereby ensuring a robust performance assessment and facilitating the development of more reliable and robust models (Gorriz et al. 2024).

### Models' evaluation

Model performance was assessed using three key statistical metrics. The MSE evaluates the mean of the squared differences among forecasted and real amounts. This metric provides insight into the magnitude of forecast errors, with larger errors being more heavily penalized. The R^2^ serves as a pointer of the model's fitting, representing the proportion of the variance in the output feature that is explained by the input parameters. Lastly, the AARE% quantifies the average of errors, presenting a clear measure of the model's exactness on a relative scale. AARE% was incorporated alongside MSE and R^2^ because it quantifies the average relative deviation between predicted and actual yields, providing a scale-independent perspective on model accuracy. This is particularly important in biochar studies, where yield values can vary substantially across different biomass types and pyrolysis conditions, making relative error a more interpretable indicator of practical performance. The combined use of these metrics ensures an inclusive assessment of model exactness, believing both the overall error extent and the model's descriptive power.

To reduce the impact of data variability, all input and target parameters were normalized prior to model training. The normalization procedure was performed according to the following equation (Cabello-Solorzano et al.):

3 $$ n^{norm} = \frac{{n - n^{\min } }}{{n^{\max } - n^{\min } }} $$

In which, the parameters *n*^min^, *n*^max^, *n*, and *n*^norm^ are the minimum, maximum, current, and normalized data.

## Results and discussion

### Sensitivity investigation

**Fig. 4:**
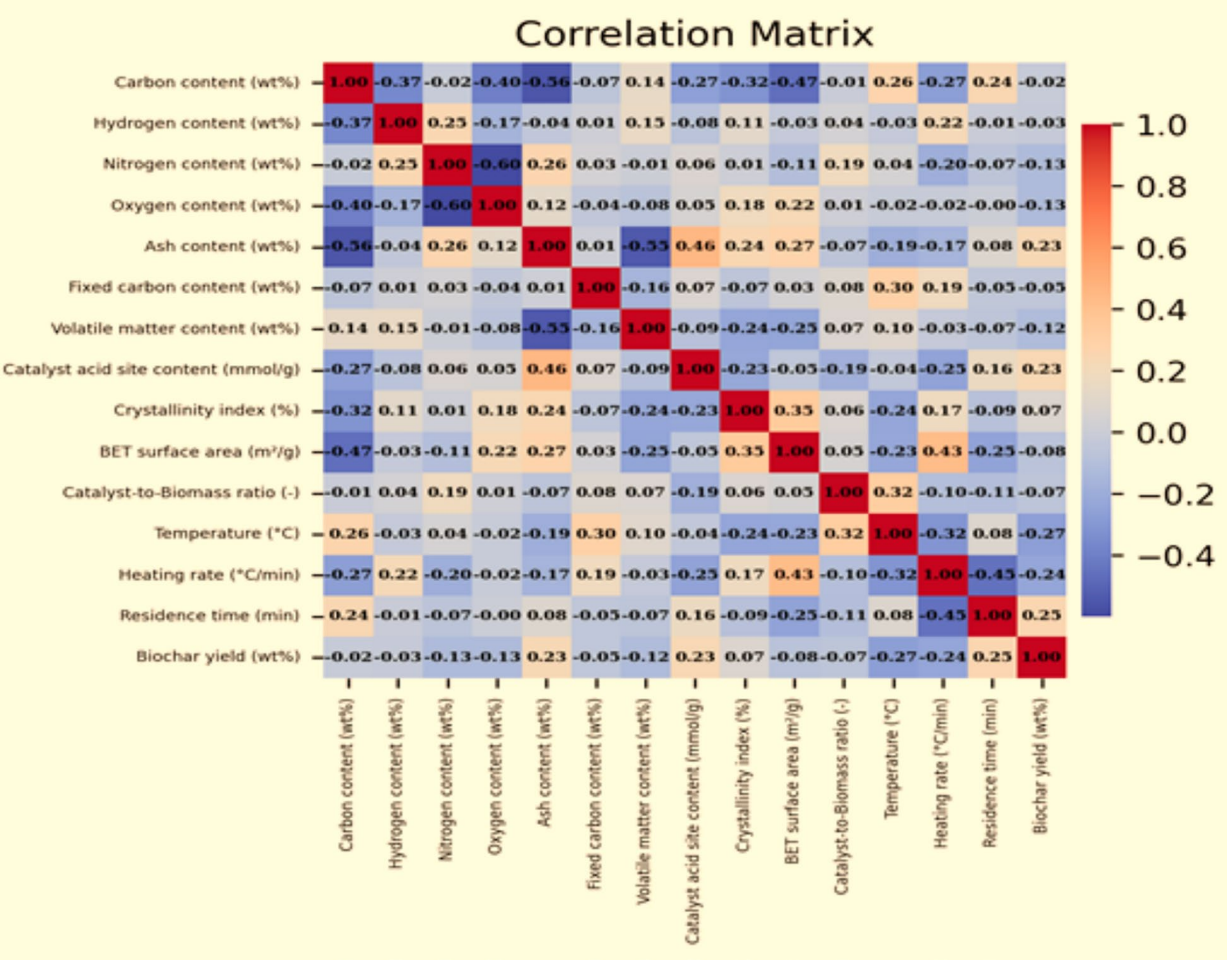
Correlation matrix of all variables analyzed, displaying the power and direction of linear relations using Pearson's correlation coefficients

The linear relationships between all variables are quantified in Fig. 4 using a correlation matrix based on Pearson's correlation coefficients. The Pearson coefficient, which ranges from − 1 to + 1, provides a measure of correlation strength and direction, with values of + 1 and − 1 indicating a perfect positive and negative relationship, respectively, and a value of 0 suggesting no linear correlation.

This matrix offers valuable visions into the interdependencies in the inputs and the target output, biochar yield. As illustrated, several variables demonstrated strong positive correlations with biochar yield, namely ash-content, residence time and catalyst acid content. In contrast, a strong negative correlation was observed for heating rate and process temperature. The remaining parameters showed a weaker influence on the yield compared to these five key variables.

The Pearson correlation matrix (Fig. 4) reveals several meaningful linear relationships among the variables. Pyrolysis temperature shows a strong negative correlation with biochar yield, consistent with the well-established trend of decreasing solid yield at higher temperatures. Residence time exhibits a moderate positive correlation with yield, reflecting its role in promoting carbonization. Among compositional variables, ash content shows a noticeable positive correlation with yield, while volatile matter displays a negative correlation. These trends provide an initial statistical indication of the parameters most likely to influence model performance, which is further confirmed by the SHAP analysis.

### Data quality corroboration

Leverage analysis is a diagnostic method used to detect unusual or influential data points in regression models. It relies on the Hat matrix, which measures how strongly each observation shapes the fitted model. The matrix is defined as (Chave and Thomson 2003):

4 $$ H = X\left( {X^{T} X} \right)^{ - 1} X^{T} $$

where *X* is the design matrix and *X*^*T*^ its transpose. The diagonal elements of *H* indicate each point’s influence; unusually large values suggest observations that may disproportionately affect model calibration. A common cutoff for identifying high-leverage points is (Ma et al. 2015):

5 $$ H^{*} = 3\left( {n + 1} \right)/m $$

with *n* representing the number of input variables and m the number of samples. Observations exceeding this threshold are flagged for further scrutiny because they may act as outliers or exert excessive influence on the regression.

**Fig. 5:**
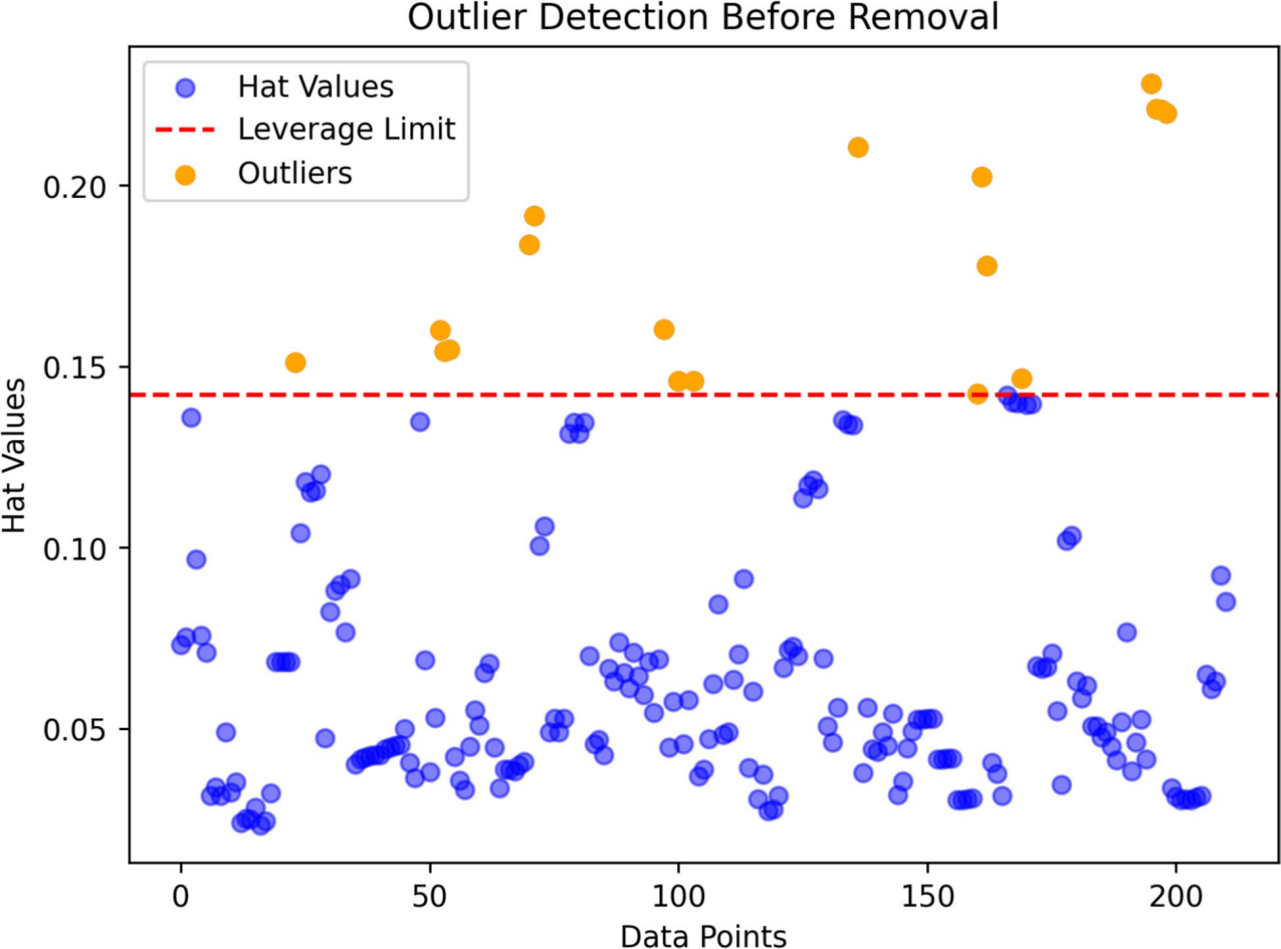
Illustrating leverage values and the corresponding outlier thresholds used to detect influential observations

These results are typically visualized using a Williams plot shown in Fig. 5, which highlights the model’s reliability region and marks suspicious points, usually fewer than 1% of the dataset, in a distinct color. By systematically identifying and evaluating such influential observations, researchers strengthen the stability, accuracy, and generalizability of their predictive models.

### Models' optimization and evaluation

Table 1 summarizes the detailed tuning of critical hyperparameters for Decision Trees (DT) using four distinct optimization strategies: TSO, ACO, ES, and BBO. Each method conducted a systematic search across predefined parameter ranges, employing both independent evaluations and cross-validation to enhance model performance. The resulting optimal configurations vary notably across techniques, reflecting the distinct search mechanisms and heuristic foundations inherent to each algorithm. The hyperparameter ranges used in this study were defined exclusively through preliminary exploratory testing, which allowed us to identify stable and meaningful parameter intervals for each optimization algorithm.

**Table 1:** Parameter ranges considered in the study and the optimized DT hyperparameters

Tuning Parameter	TSO	ACO	ES	BBO
ccp_alpha	0.00	0.06	0.02	0.01
max_depth	52	189	80	132
max_features	0.97	0.50	0.91	0.93
min_samples_split	0.03	0.03	0.02	0.01

These results highlight how the choice of optimization method directly shapes the structure and complexity of the resulting model. The comparison underscores the value of employing advanced optimization techniques for tuning DT hyperparameters, ultimately leading to more reliable and resilient predictive performance.

**Fig. 6:**
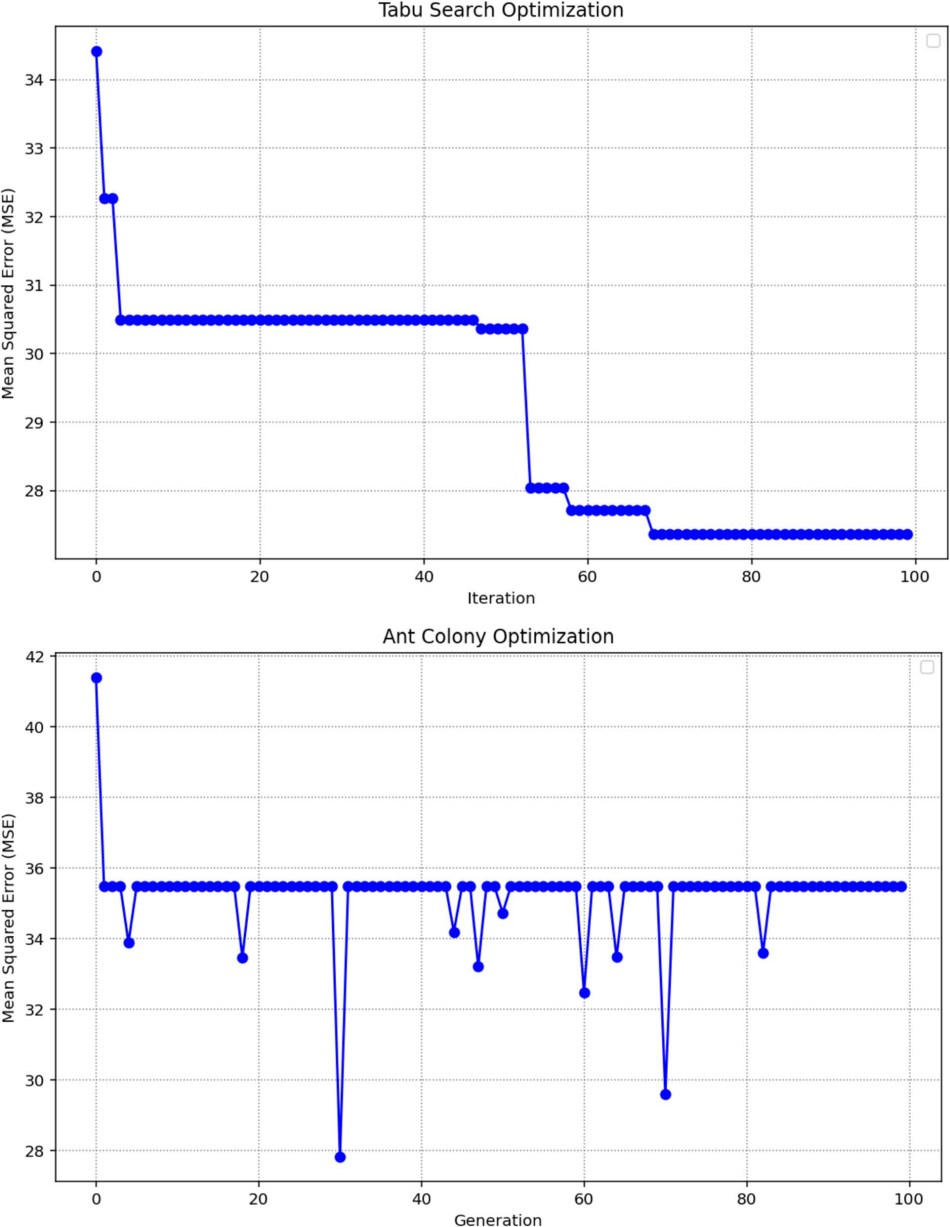

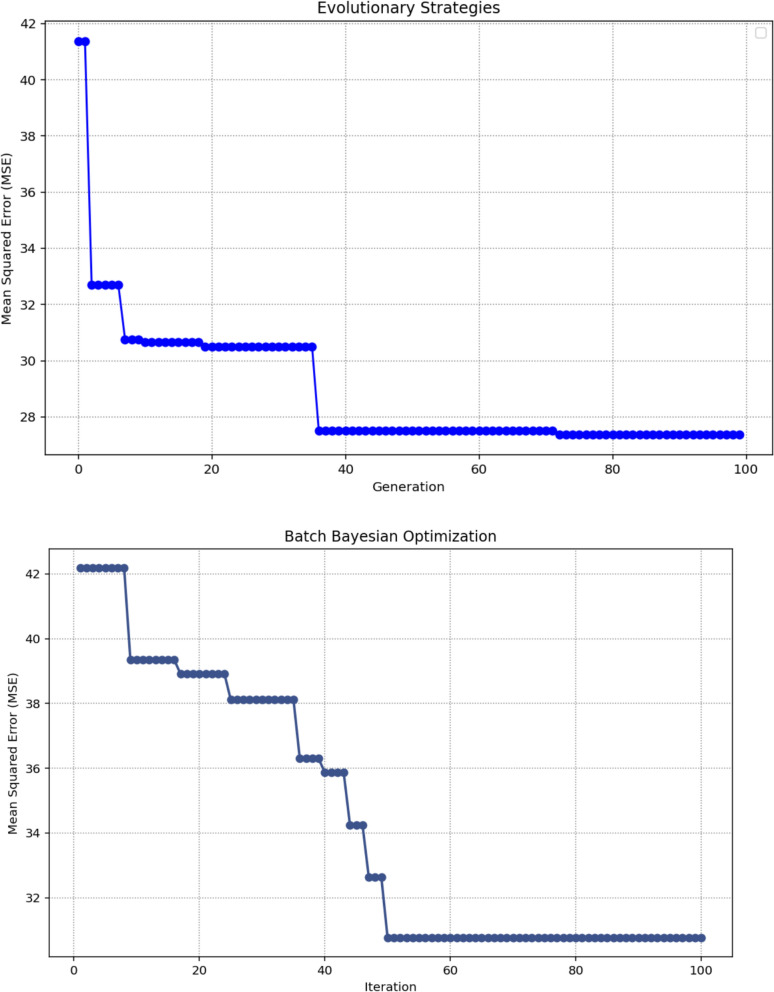
Optimizing paths of MSE over 100 iterations for each optimization method

Figure 6 presents a side‑by‑side view of the optimization progress for each algorithm by charting the MSE across 100 iterations. This comparison makes it possible to assess how efficiently each method converges toward optimal DT hyperparameters. The marked points in each curve indicate the parameter sets that achieved the minimum MSE, offering a visual reference for both solution quality and the behavior of each search process.

**Fig. 7:**
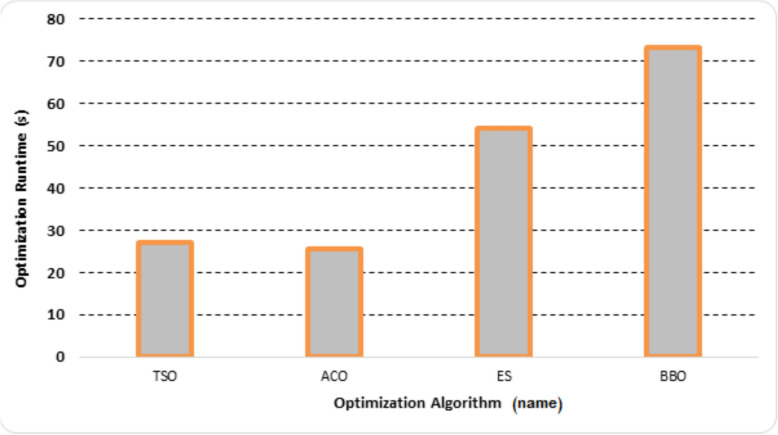
Execution time required by each optimization technique

Figure 7 shows assessment of the computing time required by each approach, recorded over 100 iterations on a standardized hardware configuration featuring an Intel Core i7-6700 processor and 16 GB RAM. While all four algorithms demonstrate broadly similar runtimes, BBO emerges as the most time-intensive, requiring approximately 73 s to complete the optimization. Conversely, ACO consistently achieves the shortest runtime, positioning it as the fastest among the tested methods.

Table 2 provides a quantitative comparison of DT models optimized using four different algorithms, revealing distinct variations in their predictive accuracy. Among the models, DT-BBO achieves the highest overall coefficient of determination (R^2^) at 0.98, followed closely by DT-ES (0.94), DT-TSO (0.94), and DT-ACO (0.90). In terms of mean squared error (MSE), DT-BBO also records the lowest total value of 1.9, outperforming DT-ES (5.4), DT-TSO (5.4), and DT-ACO (9.1).

**Table 2:** Evaluation of DT model performance, optimized using different algorithms

Optimization method	R2			MSE			AARE%		
	Train	Test	Total	Train	Test	Total	Train	Test	Total
DT-TSO	0.97	0.44	0.94	2.8	27.6	5.4	12.3	12.8	12.4
DT-ACO	0.93	0.33	0.90	6.3	33.2	9.1	19.2	14.0	18.7
DT-ES	0.97	0.44	0.94	2.8	27.6	5.4	12.3	12.8	12.4
DT-BBO	0.99	0.66	0.98	0.2	16.8	1.9	1.2	10.1	2.2

However, when considering the average absolute relative error percentage (AARE%), DT-BBO again leads with the lowest total value of 2.2%, followed by DT-ES (12.4%), DT-TSO (12.4%), and DT-ACO (18.7%). These metrics collectively indicate that DT-BBO offers the most precise overall fit across training and test sets.

Notably, DT-BBO demonstrates the highest test R^2^ value at 0.66, indicating superior generalization capability on unseen data. It also achieves the lowest test MSE (16.8) and test AARE% (10.1%) among the models, suggesting that DT-BBO provides the most reliable predictive performance on the test set.

These results underscore the importance of selecting an appropriate optimization strategy based on the specific modeling objective, whether prioritizing overall fit or test set generalization. In this context, DT-BBO emerges as a highly effective choice for robust prediction.

**Fig. 8:**
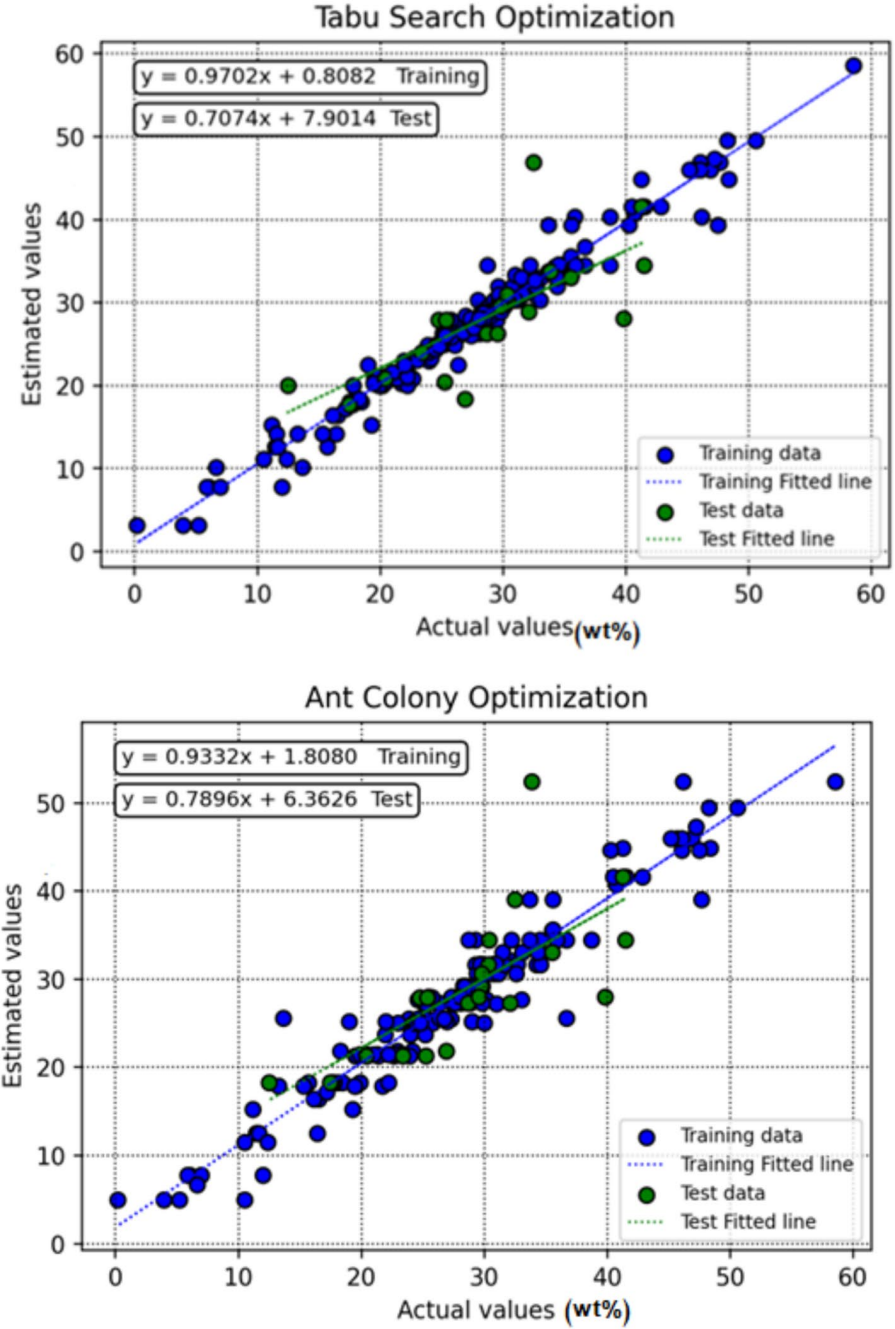

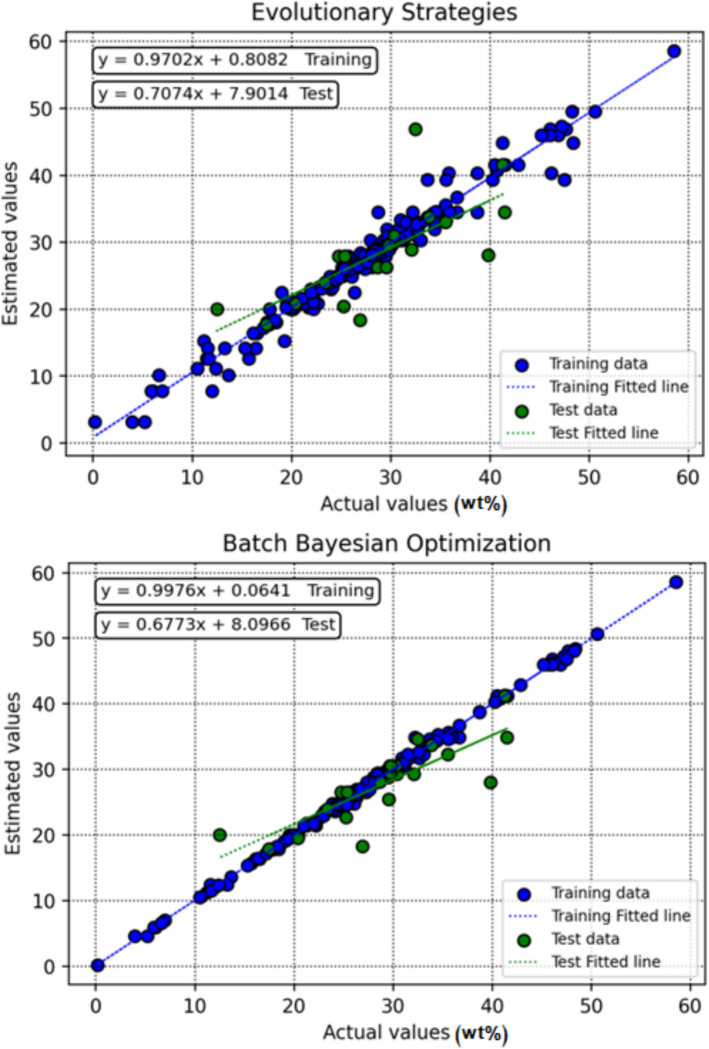
Cross-plots showing observed versus predicted amounts

Figure 8 presents cross-plots of observed versus predicted values for the different machine learning models, providing a visual assessment of prediction accuracy and overall model agreement. The degree to which each model’s outputs align with the ideal y = x line reflects its performance. Among the evaluated approaches, the DT model optimized with BBO shows predictions tightly clustered around both the best-fit regression line and the 1:1 reference line, indicating minimal scatter and strong predictive reliability.

**Fig. 9:**
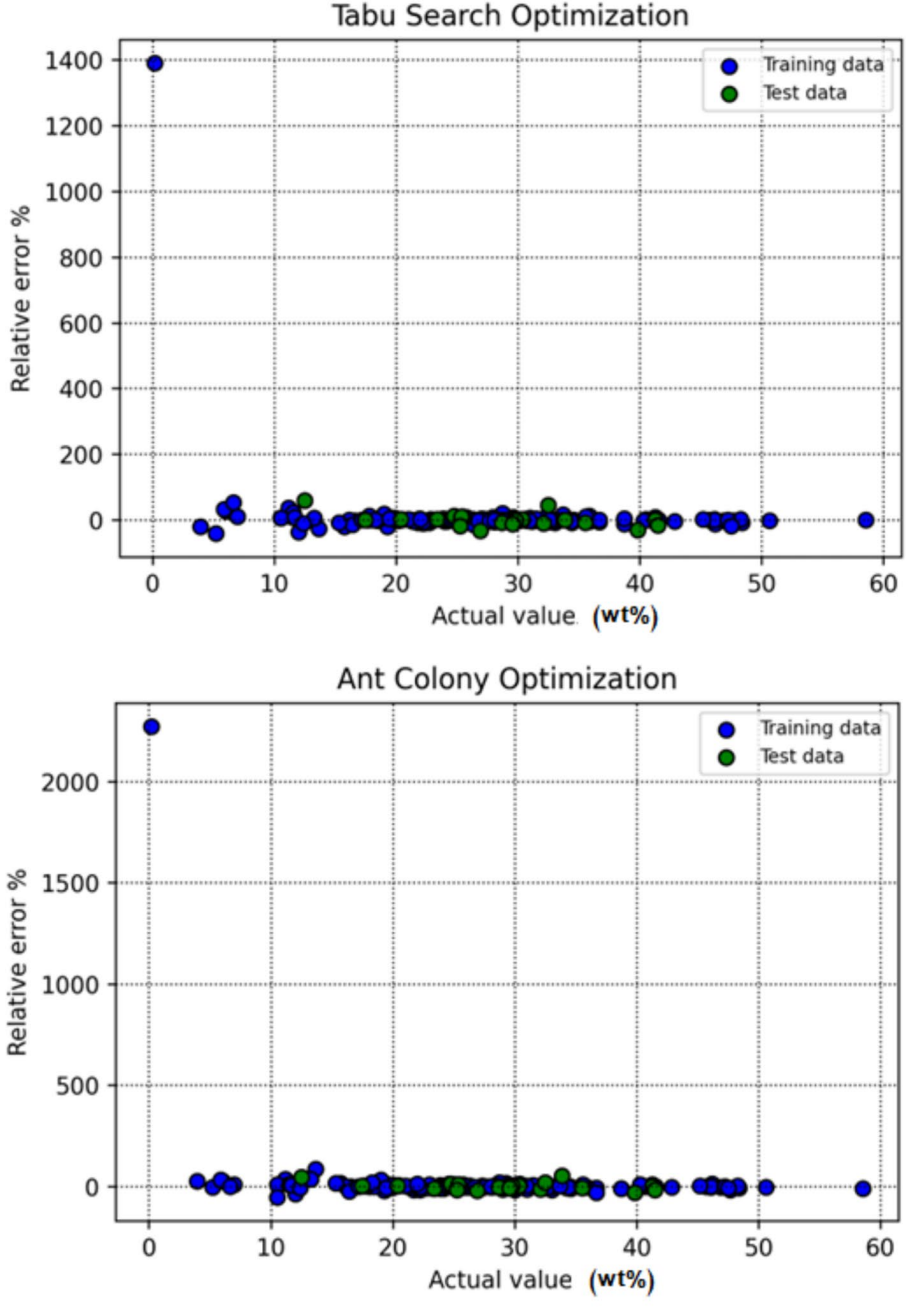

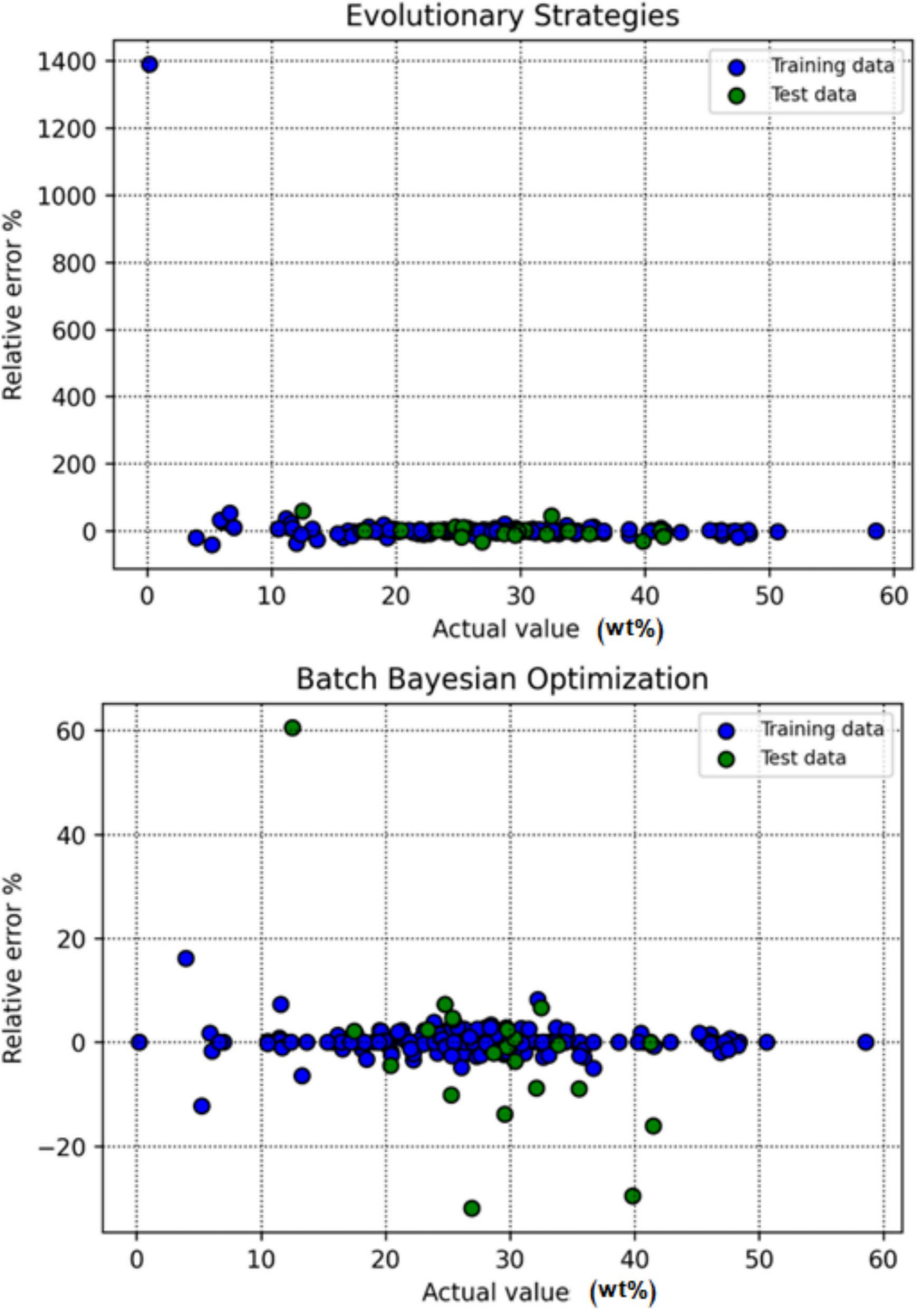
RE for individual data points in all of the models

Figure 9 displays the relative error percentage for each data point across the various modeling techniques, offering insight into error distribution and model stability. The zero-error line (y = 0) serves as a benchmark for evaluating consistency. The DT-BBO model again distinguishes itself, with RE% values concentrated closely around zero, demonstrating superior robustness and generalization compared with the other methods.

**Fig. 10:**
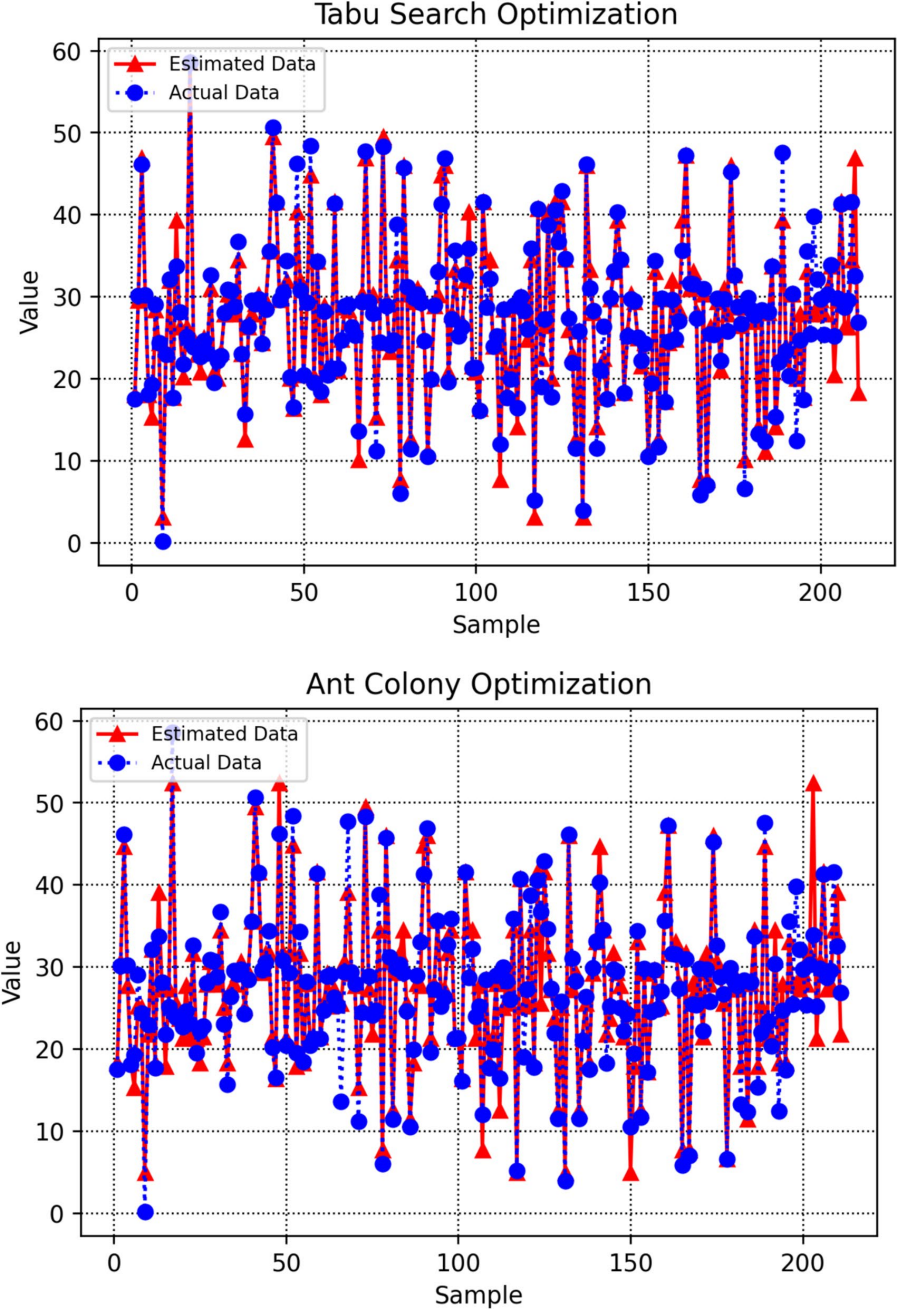

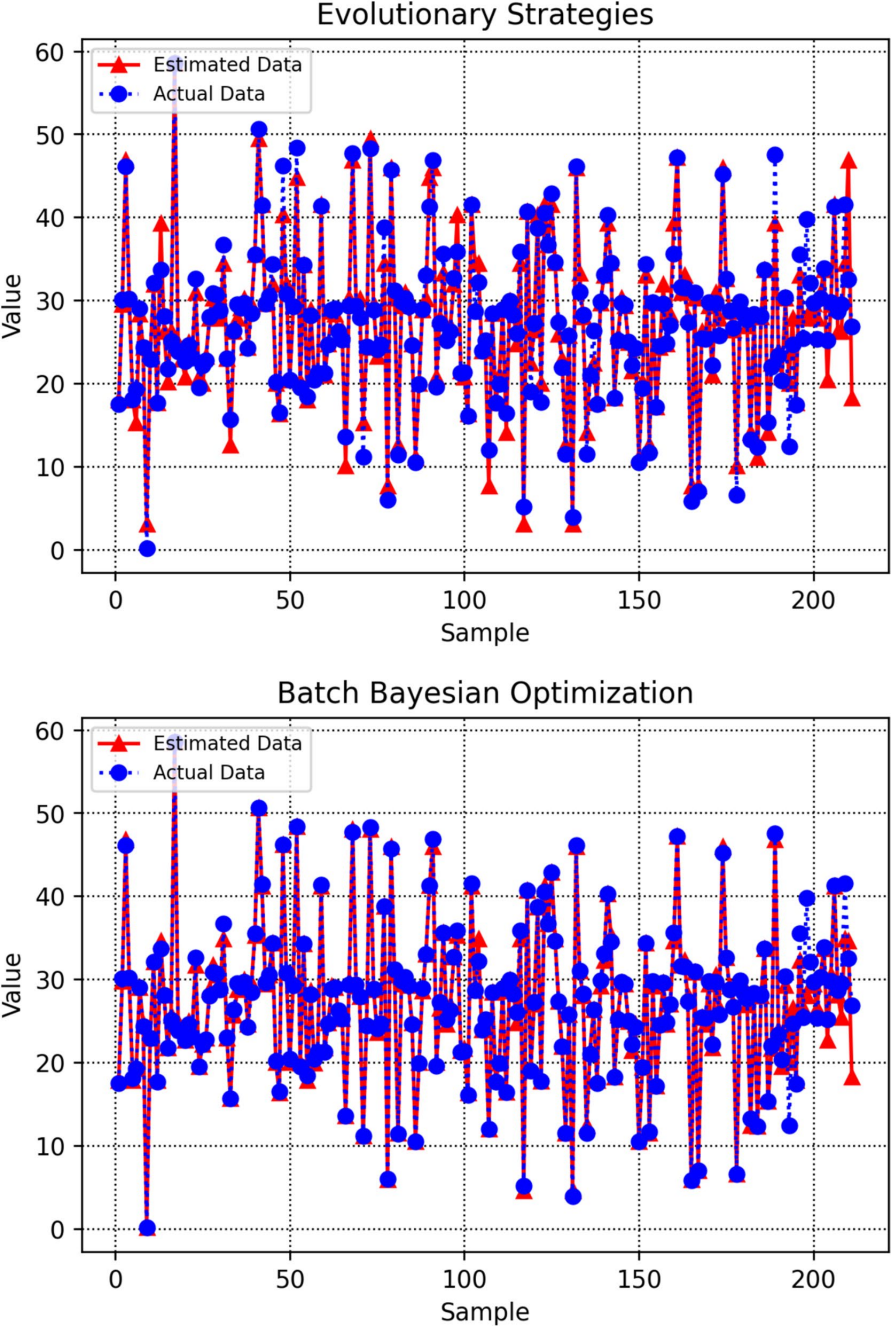
Unified comparison of observed and predicted target amounts

Figure 10 provides an integrated visual comparison of predicted and actual target values for all data points, generated by machine learning models optimized through different tuning strategies. By presenting all models within a unified diagram, the figure enables a direct assessment of how closely each model’s predictions correspond to the true values across the dataset. Among the evaluated methods, the DT model refined through BBO shows markedly lower dispersion, with its predictions clustering closely around the reference line, evidence of a more precise and well-aligned fit. This integrated visualization provides a clear and objective overview of each optimizer’s effectiveness in enhancing model generalization and predictive accuracy, allowing for intuitive comparison of performance across different tuning methodologies.

**Fig. 11:**
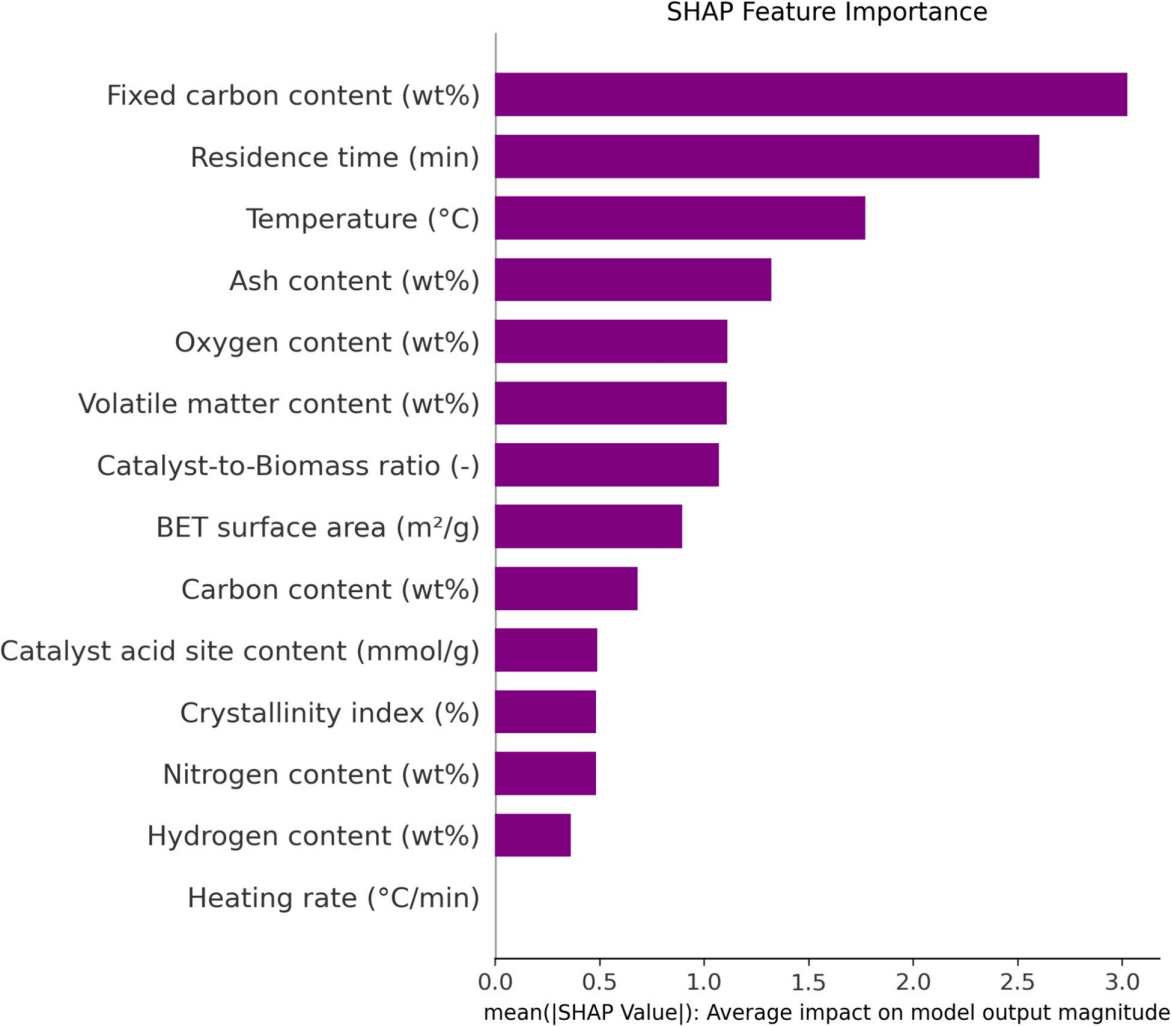
Feature importance indicated by SHAP

**Fig. 12:**
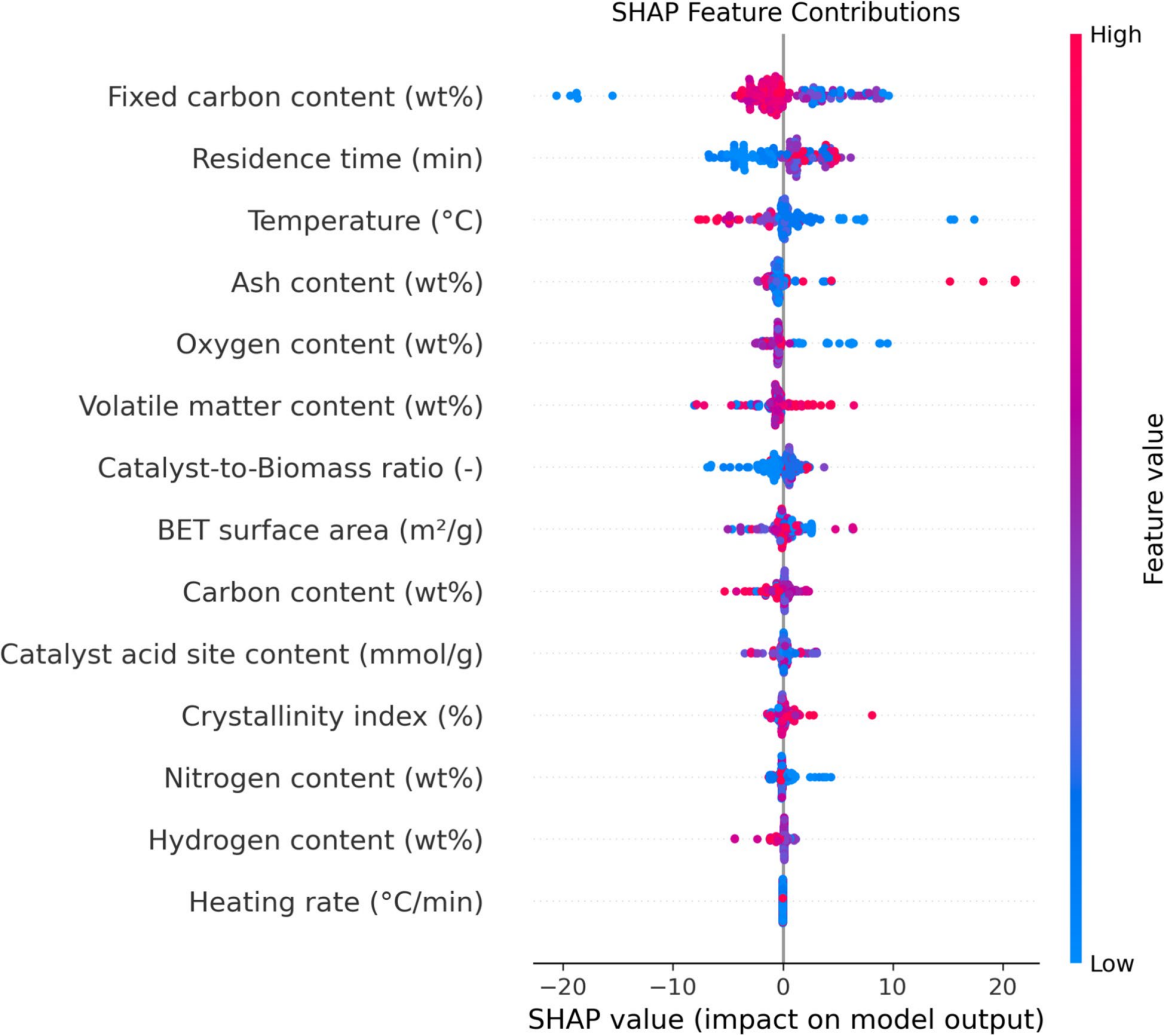
SHAP feature contributions plot

Figure 11 displays the results of a SHAP (SHapley Additive exPlanations) analysis, a method derived from idea that quantifies the involvement of each input parameter to the forecast of biochar yield. SHAPs reveal how each variable's change either surges or declines the final forecast output, offering a detailed understanding of individual feature impact. The SHAP summary plot in Fig. 12 uses a specific visual format: red specify greater feature amounts, while blue signify lower values. A point’s position on the positive or negative side of the plot indicates whether an increase in that feature’s value raises or lowers the predicted biochar yield, respectively.

The investigation tells that temperature, residence time, and ash content are the highest substantial features affecting the output. As seen in Fig. 12, high values for residence time (represented by red points) predominantly fall on the positive side, indicating a strong positive relation with biochar yield. This finding is further supported by Fig. 12, which identifies residence time as the most effective feature in the predictive model. From a chemical standpoint, this relationship is logical: a longer residence time allows for a more complete thermal decomposition of the biomass, preventing excessive loss of carbon content and ultimately leading to a higher biochar yield (Sun et al. 2017).

Conversely, the SHAP analysis for temperature indicates a negative correlation with biochar yield. The visualization demonstrates that elevated temperatures, represented by red points, are predominantly located on the negative side of the graph, signifying that higher temperatures contribute to a decrease in the predicted output.

Lastly, the SHAP analysis for ash content reveals a straight positive correlation with biochar yield. As shown in the visualization, higher ash content values (represented by red points) are predominantly located on the positive side, signifying that increased ash content leads to a greater predicted output. This is attributed to the fact that ash consists of inorganic materials that are resistant to thermal decomposition. Consequently, these materials remain in the solid phase after the volatile organic fraction has been pyrolyzed, thereby contributing to an overall higher biochar yield.

The SHAP analysis revealed that pyrolysis temperature, residence time, and ash content were the most influential features in predicting biochar yield. This ranking aligns with established pyrolysis behavior: higher temperatures typically accelerate the release of volatiles, reducing biochar yield but enhancing its stability; longer residence times promote carbonization, increasing fixed carbon content; and ash content reflects the inorganic fraction of biomass, which can catalyze or inhibit thermal decomposition depending on its composition. The model’s attribution of high importance to these features is consistent with their mechanistic roles in biomass conversion, confirming that the predictive framework captures meaningful physical relationships rather than arbitrary statistical patterns.

The SHAP analysis presented in Fig. 11 demonstrates that pyrolysis temperature, residence time, and ash content exert significantly different levels of influence on biochar yield, with temperature showing the strongest and most consistent effect. Importantly, the two most influential parameters, temperature and residence time, are also the most operationally adjustable in both laboratory and industrial pyrolysis systems, making them practical levers for process optimization. While ash content is inherently feedstock-dependent, emerging pre-processing techniques such as mineral leaching, feedstock blending, and catalytic pretreatment offer pathways to partially regulate its impact. At large scales, modern pyrolysis technologies including automated temperature-controlled reactors, continuous-feed systems, and advanced heat-transfer designs, enable precise control of these key parameters, supporting more stable and efficient biochar production. These innovations align with international sustainability efforts, particularly in carbon sequestration, renewable energy, and circular bioeconomy strategies. Therefore, future research should explore how the identified influential parameters can be integrated into scalable optimization frameworks, enabling the development of industrial pyrolysis systems that are both energy-efficient and environmentally aligned.

To contextualize the performance of the proposed DT-BBO model, a comparative analysis was conducted against previously published machine learning approaches for biochar yield prediction. Table 3 presents benchmark metrics, demonstrating that the DT-BBO framework achieves competitive or superior accuracy. In addition to its predictive strength, the model offers enhanced interpretability through SHAP analysis, distinguishing it from other black-box methods and reinforcing its practical relevance for biochar optimization.

**Table 3:** Comparative performance metrics of the proposed DT-BBO model versus previously published machine learning approaches for biochar yield prediction

Study/Model	*R*^2^	AARE%	Notes	Dataset size/biomass type
This work (DT-BBO)	0.98	2.2	Hybrid optimization with Batch Bayesian Optimization; SHAP interpretability included	211 samples; mixed biomass (agricultural residues, woody biomass, energy crops)
Zhao et al. (2025)	0.89	7.1	Applied classification-based ML methods for biochar yield prediction	150 samples; mixed lignocellulosic biomass
Liu et al. (2025) (XGBoost)	0.97	3.2	Combined ML with NPK composition prediction; interpretability analysis	180 samples; agricultural residues + woody biomass
Liu et al. (2025) (BP-ANN)	0.96	4.1	BP-ANN algorithm	180 samples; agricultural residues + woody biomass

## Conclusions

This study presents a hybrid Decision Tree framework enhanced by four optimization strategies for accurate biochar yield prediction. Among them, Batch Bayesian Optimization (DT-BBO) demonstrated superior performance, achieving R^2^ > 0.95, MSE < 2, and AARE% < 5%. SHAP recognized pyrolysis temperature, residence-time, and ash-content as the most influential, offering interpretability alongside predictive accuracy. The proposed approach contributes methodological innovation and practical relevance to sustainable biomass utilization. Future work will focus on experimental validation and broader application across biomass types to support net-zero and SDG-aligned goals.

Although the proposed DT-BBO framework demonstrates strong predictive performance, this study has several limitations that should be acknowledged. The dataset, while diverse, remains moderate in size and does not fully capture the global variability of biomass types, particularly underrepresented feedstocks such as aquatic biomass, municipal residues, and region-specific agricultural wastes. Additionally, some influential parameters such as ash content are inherently feedstock-dependent and may require more detailed characterization in future datasets. To enhance generalizability, future work should focus on expanding the dataset with broader geographic and compositional diversity, integrating real-time industrial pyrolysis data, and exploring hybrid optimization–interpretability frameworks for large-scale deployment. Advancements in automated reactor control, continuous-feed pyrolysis systems, and sensor-driven monitoring also present promising avenues for translating these findings into scalable, industry-aligned biochar production strategies.

## Data Availability

Data is available on request from the corresponding author.
